# Year of the face mask: do’s and don’ts during exercise

**DOI:** 10.17159/2078-516X/2020/v32i1a8615

**Published:** 2020-01-01

**Authors:** DC Janse van Rensburg, L Pillay, S Hendricks, JA Hamuy Blanco

**Affiliations:** 1 Section Sports Medicine & Sport Exercise Medicine Lifestyle Institute (SEMLI), Faculty of Health Sciences, University of Pretoria, Pretoria, South Africa; 2 International Netball Federation, Manchester, UK Medical Board Member, UK; 3 Division of Exercise Science and Sports Medicine (ESSM), Department of Human Biology, Faculty of Health Sciences, University of Cape Town; 4 Carnegie Applied Rugby Research (CARR) centre, Institute for Sport, Physical Activity and Leisure, Leeds Beckett University, Leeds, United Kingdom

**Keywords:** COVID-19, masks, exercise, physical activity, corona virus, prevention, health

## Abstract

The COVID-19 pandemic causes widespread anxiety and uncertainty regarding disease transmission. In many countries people are obliged to wear a face mask in public spaces. Individuals involved in sports participation at any level need to make informed decisions on wearing a face mask during exercise. Currently there is no scientific evidence on what to advise regarding the safety of wearing a face mask during exercise, or what type of mask to use. This short report aims to answer these questions in a structured and practical way.

The coronavirus disease 2019 (COVID-19) is a worldwide pandemic. The lockdown levels were recently changed in South Africa and the period for outdoor exercises, such as cycling and jogging, is presently from 4 am to 9 pm. Exercise is currently restricted to not more than four people together. Even so, people choosing to exercise still have the potential of exposure to COVID-19-containing droplets from those around them. While exercising, one must observe the behaviour of others in order to protect oneself, especially from those exercising nearby, to ultimately stop the spread of the virus.

To safeguard the health of all, the key steps to prevent the spread of the disease include the frequent washing of hands with soap, social distancing and covering the face with a mask/buff to limit virus-laden droplets being transmitted. Indeed, the government has imposed regulations about wearing masks in public spaces. Guidelines from the National Institute for Communicable Diseases (NICD) and the Department of Health (DoH) refer to general public health with nothing specifically related to exercise. No real evidence exists for the prevention of COVID-19 during exercise and most of the recommendations to date are deductions from other areas of study, specifying how these can be clinically and practically applied.

## Discussion

### How do these protective behaviours impact exercise?

- COVID-19 disease is a highly transmissible and pathogenic viral infection caused by the severe acute respiratory syndrome coronavirus 2 (SARS-CoV-2). Human-to-human spreading of the virus occurs when one comes into contact with an infected person, mainly via small respiratory droplets or aerosols passed on through coughing, sneezing or even exhaling. These droplets can enter the lungs via inhalation through the nose or mouth. ^[1]^
- During aerobic exercise, the rate and depth of breathing increases to ensure that more oxygen is absorbed into the blood and carbon dioxide is removed. Because COVID-19 penetrates the lungs via inhalation through the nose or mouth, it is necessary to be extra careful during exercise. How humans interact during activities such as running is also worth considering. Using wind tunnel computer imaging, a recent simulation study by Blocken et al. (2020) from the Eindhoven University of Technology showed that the potential aerodynamic effect of respiratory droplet transfer is greatest when running in a straight line with one person following behind another. In this case, it is the person directly behind the others who is the most exposed.^[2]^ While this was a simulation study, the authors suggested: (1) running side by side with a two metre distance between runners; however, this is virtually impossible on public roads; (2) running at least five metres (cycling 10 metres) behind the person in front– which may become a problem if there are many people nearby; (3) running with a mask or buff on as this will reduce the droplets that are aerolised by the virus; and (4) staggered running/cycling especially when passing someone.

### Can one run with a mask and if so, which kind of mask is best?*

This is most definitely possible. Selecting a face mask becomes a balancing act between maintaining comfort, breathability and concerns about infection control.

- Cloth face masks or ‘buffs’ made of cotton material are best. In a recent publication, Greenhalgh et al. (2020) reported that using cloth face masks help to prevent droplets from being transmitted, thereby limiting community spread.^[3]^ The mask is meant to protect the individual. Even a dual layer buff will be suitable and enable one to breathe while exercising. The middle layer (in three layer masks) is often made from synthetic materials which may make masks hotter and harder to breathe through.
- Theoretically, moisture-wicking material will be more comfortable (i.e. more breathable), but at the cost of less effective containment of droplets due to the tiny spaces in the fabric that allow moisture removal. Moisture-wicking depends on capillary action, namely the movement of a liquid (in this case, exhaled breath) through the small spaces within the fabric. The job of the moisture-wicking fabric is to rapidly move ’liquid’ to the outer surface of the fabric and to dry quickly to prevent saturation of the fabric. Most moisture-wicking fabrics are synthetic, e.g. polyester and nylon.
- It is preferable to use a cloth mask with a maximum of two layers. If the seal is too tight around the sides of the face there will be less movement of air.
- As previously mentioned, buffs are usually made from synthetic, lightweight material designed to reduce heat build-up. They are also open at the bottom, allowing for better air flow. This can be good from a comfort point of view, but is perhaps less effective for infection control.
- Vented masks, if fitted correctly, have a one-way valve that protects the wearer by allowing for easier exhalation, the reduction of the build-up of carbon dioxide, and the prevention of humidity. The problem is that it only filters inhaled breath and not exhaled breath. Although a vented mask is more comfortable for the wearer, it, unfortunately, does not contain the droplets.
- There is a worldwide shortage of personal protective equipment for healthcare workers and others on the frontline. Respirator masks such as the FFP1, FFP2 and N95 should be reserved for that purpose, and for use in the other specific working environments that require them. The general public should be using cloth face masks.

*Specific to South African regulations: RSA Recommended Guidelines: Fabric Face Masks Manufactured by South Africa’s Clothing and Textile Manufacturing Industry for General Public Use. 9 April 2020 https://www.nicd.ac.za/wp-content/uploads/2020/04/RSA_Recommended-Guidelines-for-Masks_Summary_FINAL09.04.2020_for_distribution.pdf

### Taking care of the cloth mask

- Wash the mask regularly with soap and water.
- Dry the mask thoroughly and then preferably iron it.

### Is it dangerous to exercise with a cloth mask?

- If a mask is not cleaned regularly, it may be contaminated with the novel coronavirus and also other viruses or bacteria. Always have an extra mask at hand while the other is being washed and dried.
- Proper handling of the mask is vital. Never touch the exposed front of the mask as this is where the highest concentration of potential viruses and bacteria can be found.
- A mask must be breathable. This is subject to the thickness of the material and the tightness of the seal on the face. A mask theoretically increases the breathing effort and causes an accumulation of CO^2^.
- The more breathable the material, the less likely it is for CO^2^ to accumulate. When running it is necessary to breathe in oxygen and breathe out carbon dioxide without carbon dioxide accumulating, which could cause fainting.
- Cloth masks or buffs are likely to get wet during exercise, due to the accumulation of moisture from exhaled breath. Breathing through damp cloth is more strenuous. This can be more of an issue in hot and humid conditions, so anticipate this and act accordingly. Consider taking a second mask/buff during an exercise session to change into once the original one is saturated with moisture. However, this can also be tricky as one should try to touch one’s face as little as possible
- If medical conditions exist, such as obstructive lung disease, bullous lung disease, other lung diseases or heart diseases, it will be best to get a doctor's opinion before running with a mask as this may worsen any medical condition.^[4–6]^

### Is it possible to exercise at any intensity with a cloth mask?

It will be possible to exercise easily with a mask while doing low-intensity exercise. However, with moderate/high-intensity exercise, it may become difficult to exercise with a mask and could pose dangers as mentioned above.

Exercising at moderate or high intensities, may bring on the abovementioned medical risks. If the intention is to exercise at these intensities, it should be done outside at home and not wearing a mask. However, this will require specific clearance from the government at Level 3 of the risk-adjusted approach of lockdown.

### Important reminders

- There is unfortunately an inverse relationship between protection and breathability with regards to the wearing of masks while exercising.
- It is important not to exercise at all when feeling ill. Consult with a doctor, get treated accordingly, and obtain their medical permission to exercise. If attempting to exercise with ’flu’ symptoms, there is a potential that this viral infection may affect the heart, causing myocarditis (infection/inflammation of the heart muscle) that may lead to sudden cardiac death. Since the novel coronavirus is new, not much is understood about it yet and it must be assumed that the same problems can occur as when exercising with ‘flu’.
